# Top-level leaders and implementation strategies to support organizational diversity, equity, inclusion, and belonging (DEIB) interventions: a qualitative study of top-level DEIB leaders in healthcare organizations

**DOI:** 10.1186/s13012-023-01319-7

**Published:** 2023-11-07

**Authors:** Tory H. Hogan, Brian P. O’Rourke, Eddie Weeks, Geoffrey A. Silvera, Seongwon Choi

**Affiliations:** 1 The Division of Health Services Management and Policy, The College of Public Health, The Ohio State University, 1841 Neil Avenue, Columbus, OH 43210 USA; 2 The Department of Health Services Administration, The School of Health Professions, The University of Alabama at Birmingham, School of Health Professions Bldg., 1716 9th Avenue South, Birmingham, AL 35233 USA; 3 The Department of Management, The College of Business and Economics, California State University, Los Angeles, 5154 University Dr, Los Angeles, CA 90032 USA

**Keywords:** Healthcare delivery system, Diversity equity and inclusion, Top-level leaders, Health equity, Organizational interventions

## Abstract

**Background:**

The Black Lives Matter movement and COVID-19 pandemic motivated the wide-scale adoption of diversity, equity, inclusion, and belonging (DEIB) initiatives within healthcare organizations and the creation of DEIB top-level leader positions. The next step is to understand how these leaders contribute to the implementation of DEIB interventions, a task with notable salience due to not only the historical difficulties associated with DEIB strategy execution, but also the substantial evidence that leadership plays a significant role in implementation processes. Therefore, the objective of this qualitative study is to understand the role of top-level DEIB leaders in the implementation of healthcare organizational DEIB interventions.

**Methods:**

A qualitative research approach which used an in-depth semi-structured interview approach was employed. We conducted thirty-one 60–90-min semi-structured interviews with DEIB top-level leaders between February 2022 and October 2022 over Zoom. An iterative coding process was used to identify the key implementation strategies and activities of DEIB top-level leaders.

**Results:**

Interviewees were mostly Black, majority female, and mostly heterosexual and had a variety of educational backgrounds. We identified the DEIB top-level leader as the DEIB strategy implementation champion. These leaders drive five DEIB implementation strategies: (1) *People,* (2) *Health Equity*, (3) *Monitoring and Feedback*, (4) *Operational Planning and Communication*, and (5) *External Partners*. Within these, we identified 19 significant activities that describe the unique implementation strategies supported by the DEIB top-level leaders.

**Conclusions:**

To move toward sustained commitment to DEIB, the organization must focus on not only establishing DEIB interventions, but on their successful implementation. Our findings help explicate the implementation activities that drive the DEIB initiatives of healthcare organizations and the role of DEIB leaders. Our work can help healthcare organizations systematically identify how to support the success of DEIB organizational interventions.

**Supplementary Information:**

The online version contains supplementary material available at 10.1186/s13012-023-01319-7.

Contributions to the literature

- Provides a detailed examination of how DEIB top-level leaders implement DEIB interventions that are a part of healthcare delivery organization’s strategic plans.
- Identifies the top-level DEIB leader as the champion of implementing organizational DEIB interventions.

## Background

In recent years, the Black Lives Matter movement and the COVID-19 pandemic set in motion the acknowledgment by healthcare organizations (HCOs) that they must address health inequities, a lack of diversity among their workforce, and persistent problems related to workplace climate. Simultaneously, the research community has acknowledged consistent patterns of racial and ethnic health and healthcare disparities within the US healthcare system and called for HCOs to not only stand against these inequities, but to examine *how* to eliminate inequities through organizational change [1–4]. As a part of this, hospital boards and healthcare executives of HCOs have made diversity, equity, inclusion, and belonging (DEIB) an organizational priority of the delivery organization and have adopted DEIB organizational interventions. DEIB organizational interventions describe planned, systematic organizational efforts to make changes to address DEIB issues and improve the organization’s performance. Additionally, top-level executive roles have emerged to take primary responsibility for supporting the formulation and implementation of these DEIB organizational interventions [5]. Organizations often choose titles for these positions such as Chief Diversity Officer, Chief Health Equity & Diversity Officer, and Chief Inclusion, Diversity & Health Equity Officer.

DEIB organizational interventions, sometimes referred to as “DEIB strategies,” frequently include a combination of human resources and patient-focused efforts. Human resource strategies include commitments to fairness in hiring, compensation, and promotion; DEIB staff training; addressing disparities in the employee experience; and creating a climate of inclusion [4, 6]. DEIB clinical strategies focus on providing equitable care through the improvement of disparities in access, care, treatment, and outcomes; research efforts focus on driving advancements in health equity, engagement with communities who traditionally have not been represented, and a structured and intentional approach to supplier diversity that can better support the diverse patient population [7, 8]. Following the selection of one or more of these DEIB strategies, organizations begin implementation, commonly referred to within HCOs as “execution” or “adoption.”

Implementation has long been a struggle within organizations across industries; therefore, it is important to understand the process and the factors that contribute to it [9]. In fact, it has been argued that implementation is more difficult than the formulation and planning of organizational interventions [10]. There are a multitude of factors (e.g., communication, commitment, systems, leadership, human resource management practices, organizational structure, and administrative processes) [11] that play a role in the process of successful implementation of organizational interventions. Within these factors, leadership and the activities of top-level leaders are significant at various stages of the implementation process and have been identified across a multitude of strategy implementation models [12, 13]. Considering HCOs’ commitment to DEIB, the emergence of DEIB top-level leadership positions, and the large body of work documenting the challenges associated with strategy implementation, little is understood about how these new top-level leaders (DEIB leaders) steer the implementation of DEIB interventions.

Implementation science frameworks identify the organizational context as a critical factor or construct of implementation success and suggest that leadership can support and improve the implementation of evidence-based practices within the organizational context [14–18]. Empirical studies have supported the association between leadership and implementation in the healthcare sector, as it can serve to motivate, influence, and enable individuals and groups [13, 19, 20]. Beyond this, implementation science has identified that leadership across different levels (top, middle, and front-level) each has important roles to play in supporting implementation activities. Top-level leaders determine organizational policies, allocate resources to support processes and establish organizational practices, and coordinate across levels of the organization. Understanding the role of top-level leaders in successfully implementing DEIB interventions is especially important as the emergence of top-level DEIB leaders in HCOs has become more common.

In addition to the importance of top-level leadership in the implementation of organizational interventions, the implementation of DEIB initiatives poses particular challenges due to the lack of robust evidence, as well as structural barriers to alleviating structural inequities in society [21–23]. As Brownson et al. describe, “While health equity is a high priority for many public health organizations, there is sparse empirical data on the organizational commitment to equity issues and how that commitment is operationalized” (p. 11) [21]. Thus, there is a clear need to contextualize and articulate how organizations deploy resources to support the structures and processes to systematically address DEIB issues. Enhancing understanding of this issue will contribute to a systematic approach to evaluating what specific implementation strategies support DEIB organizational interventions. It also supports a broader understanding of the role of HCOs in improving health outcomes within the US healthcare delivery system. The increasing prominence of DEIB top-level leaders across US HCOs sends an important signal that organizations are approaching DEIB in new ways. It is urgent to study this phenomenon and identify the role of organizations in supporting a more inclusive and equitable healthcare delivery systems.

To move the implementation science field toward this broad goal, this qualitative study aimed to address a gap in the literature around the implementation strategies that HCOs adopt to support organizational-wide interventions related to DEIB. We achieve this aim through two study objectives: First, we examine the role of healthcare delivery organization’s DEIB top-level leaders in the implementation of organizational DEIB interventions. Second, we identify an empirically driven list of implementation strategies that the DEIB top-level leader employs within HCOs. This approach focuses on understanding how DEIB leaders support the organization’s adoption of DEIB interventions. We built this knowledge by conducting thirty-one 60-min, semi-structured interviews with top-level DEIB leaders in US HCOs or DEIB subject matter experts and used a hybrid inductive-deductive analytical approach to evaluate and identify DEIB top-level leaders’ strategy implementation activities.

## Theoretical framework

Given the complex nature of organizational DEIB interventions, and the need to identify and understand the specific modalities that support this type of work in organizations, we conceptualized the activities of the DEIB leaders and DEIB organizational interventions using a framework which is based on strategic management theory, specifically what the field of strategic management refers to as “strategy implementation.” Within the field of strategic management, strategy implementation describes the processes that support an organization’s ability to do the intervention. Implementation science refers to these as “implementation strategies.” Additionally, strategic management describes the specific organizational intervention and processes related to determining these interventions as the “organizational strategies.” Regardless of the differences in terminology between fields, our study utilized these strategic management concepts as the foundation of our framework due to their ability to address and describe the process organizations go through when identifying strategies associated with organizational-wide interventions. More specifically, we utilize a conceptual framework identified by Noble [24] and Okumus [25, 26] in the field of strategic management that incorporates elements of the role of leaders in strategy implementation. In the implementation science field, one would say that this conceptual framework explicates implementation strategies.

### Strategy implementation framework

Research has identified the importance of leadership and its relation to organizational interventions and implementation strategies. For example, scholars have identified how leaders influence employees and implement organizational change through the process of adopting organizational interventions as a critical element of successful implementation. We build off the concepts of leaders as a part of the process identified by Noble [24] and argue that HCOs utilize a top-level leader as the “champion” of DEIB implementation processes. As the champion, the top-level leader coordinates resources, collaborates with organizational leaders, identifies the appropriate functions and stakeholders within each strategy component, and tracks and communicates the movement toward implementation.

With a top-level leader as the “champion” of the DEIB strategy implementation, we utilize the *Strategy Implementation Framework* to provide a context for understanding the detailed activities which make up the implementation process of DEIB interventions. The *Strategy Implementation Framework* by Okumus [25, 26] identified four groupings of factors that impact implementation in organizations: *Strategic Content, Strategic Context, Operational Process*, and *Outcome*. The *Strategic Content* describes the plan for organizational interventions, and how and why it is developed. Health delivery systems regularly (e.g., annually, every 3–5 years) go through a strategic planning process that includes identifying organizational-level interventions needed to achieve HCO goals. For example, in relation to DEIB work, this could include interventions that will improve patient experience among minoritized populations, increase the representation of minoritized populations in their workforce, achieve a more inclusive culture, address social determinants of health during patient care, etc. The *Strategic Context* describes internal and external factors which can incentivize organizations to adopt strategies and interventions or to support their successful implementation. There are a multitude of factors within the healthcare delivery system which create internal and external factors that promote the adoption of DEIB strategies and interventions. These external factors could include the Black Lives Matter movement or COVID-19 pandemic, or pressures from health reform such as population health policies which aim to improve health outcomes for specific populations. Internal factors could be related to the high turnover among employees from minoritized populations or feedback from employee resource groups. The *Operational Process* refers to the implementation activities within the organizations that enable the change the strategic plan requires. The DEIB top-level leader is the champion of the processes that are needed or a part of implementing the DEIB strategies established in the *Strategy Content* domain. This includes the development of human capital, management systems, resources and processes, communication systems, and feedback and monitoring mechanisms. The last factor is *Outcome*, and this describes the intended and unintended results.

In this study, we focus on the five *Operational Processes* of strategy implementation: (1) Operational Planning refers to the processes associated with initiating and planning for the new strategy; (2) Resource Allocation are processes which ensure all the necessary resources (time, human capital, financial, skills) are activated and available to be utilized during the process of implementation; (3) People refers to the processes of hiring, recruiting, training, and activating the human capital needed to execute a strategy; (4) Communication refers to a multimodal approach of formal communication methods and informal methods in which strategies are embedded into existing systems, e.g., incentive plans; and (5) Control and feedback refer to the mechanisms for monitoring and measuring the organization’s results of the strategy implementation. These five implementation processes conceptualize the DEIB leader’s role in implementing DEIB strategies/interventions.

Building on the previously described concepts of Noble and Okumus, we propose a theoretical framework of DEIB implementation strategies that identifies organizations’ efforts to establish DEIB interventions, which then results in the identification of a top-level leader as the champion of the DEIB implementation strategies. As the champion, top-level leaders manage and lead the implementation strategies which drive the DEIB intervention outcomes. We use this framework in the following ways:

1. To understand and justify the relationship between the adoption of DEIB strategies/interventions in HCOs and the top-level DEIB leaders.
2. To act as a practical guide to systematically explore the role of the DEIB leader in DEIB strategy implementation.
3. To provide a theoretical underpinning used during axial coding and the identification of DEIB leader implementation strategies/processes.

## Methods

We employed a qualitative research approach using in-depth semi-structured interviews. This approach was adopted to facilitate an in-depth and detailed analysis of the specific ways DEIB leaders participate in the implementation of healthcare delivery organization’s DEIB interventions, as well as allow for a detailed analysis of the complexity associated with implementing DEIB interventions [27]. We conducted 31 semi-structured interviews between February 2022 and October 2022 over Zoom with healthcare senior leaders whose primary responsibility was related to DEIB in healthcare delivery organizations. Interviews lasted approximately 60–90 min. A completed copy of the Consolidated criteria for reporting qualitative research (COREQ) was completed and is available in Additional file 1 (“ISSM COREQ Checklist” [28]).

### Participant eligibility, recruitment, and data collection

Individuals were eligible to participate if they met any of the three following criteria: (1) served or previously served as a top-level leader in a healthcare organization whose primary responsibility was related to diversity, health equity, inclusivity, and/or belonging. These individuals generally held titles such as Chief Diversity Officer, or a variation of this. Twenty-seven of the 31 participants met this criterion; (2) worked as a senior executive of DEIB healthcare consulting firms and primarily provided advisory services to HCOs to support the adoption of organizational-wide DEIB interventions and support large-scale implementation of DEIB interventions in HCOs: 2 of the 31 participants met this criterion; (3) was a senior member of the top-level DEIB leader’s team when this leader was not available—this accounted for 2 of the 31 participants as well. We verified that the participants for this criterion would be able to answer questions about the DEIB top-level leader. These inclusion criteria ensured the study team would be able to interview individuals who functioned within the top-level of organizations or could speak to the activities of top-level DEIB leaders. Our decision to include senior executives from DEIB healthcare consulting firms ensured that the study findings incorporated the experience of subject matter experts in the field who serve as leaders in the field of organizational-wide DEIB interventions, and ensured the study gained a broad perspective of the DEIB roles in healthcare, as these individuals interact and provide subject matter expertise to many DEIB top-level leaders across a variety of HCOs. While we acknowledge that senior executives from DEIB healthcare consulting firms do not have the same understanding of implementing DEIB organization-wide interventions as other participants in our study, these participants were able to provide key subject matter expertise on the implementation process based on working closely with DEIB leaders.

The recruitment process began with the study team brainstorming a list of potential participants that individual team members knew through professional networks. We recruited these potential participants via email, which included recruitment materials describing the study purpose and Institutional Review Board (IRB) information, as well as a request to participate in one sixty-minute interview. The study team also asked participants if they knew of anyone who may be interested in participating in the study. Among the 31 participants, 15 were employed at Community Health Systems, 8 at Academic Medical Centers (AMCs), 4 at Children’s health systems, 3 at consulting firms, and 1 at a government health system. Interviews were conducted by four researchers. The lead author is a subject matter expert on qualitative research methods, having published numerous peer-reviewed articles using qualitative methodologies, and regularly giving invited lectures and presentations at international conferences on qualitative research methods topics. To ensure consistency across interviewers, prior to starting data collection, the team met and reviewed the interview guide and developed consensus regarding ways to develop trust and solicit in-depth responses from study participants. Additionally, three researchers each observed an interview conducted by the lead authors to learn the agreed upon approach. All interviews were conducted using Zoom, with the team keeping a record of the Zoom transcript and the audio recording. Transcripts were cleaned to ensure transcription accuracy and readability. The study was approved by the institutional review board of The Ohio State University (IRB #2021B0369).

### Interview guide

To develop our interview guide, we utilized a multi-step process with included reviewing previous literature on DEIB leaders in healthcare delivery organizations, formulating a preliminary semi-structured interview guide, pilot testing our interview guide with individuals who fit the participation criteria, and revising the preliminary interview guide for clarity and completeness in response to feedback from pilot testing. The interview guide also included open-ended demographic questions, allowing the participant to self-identify characteristics such as race, gender, sexual orientation, and educational background. A copy of the interview guide is available in Additional file 2 (“Interview guide”).

### Analytical approach

**Fig. 1:**
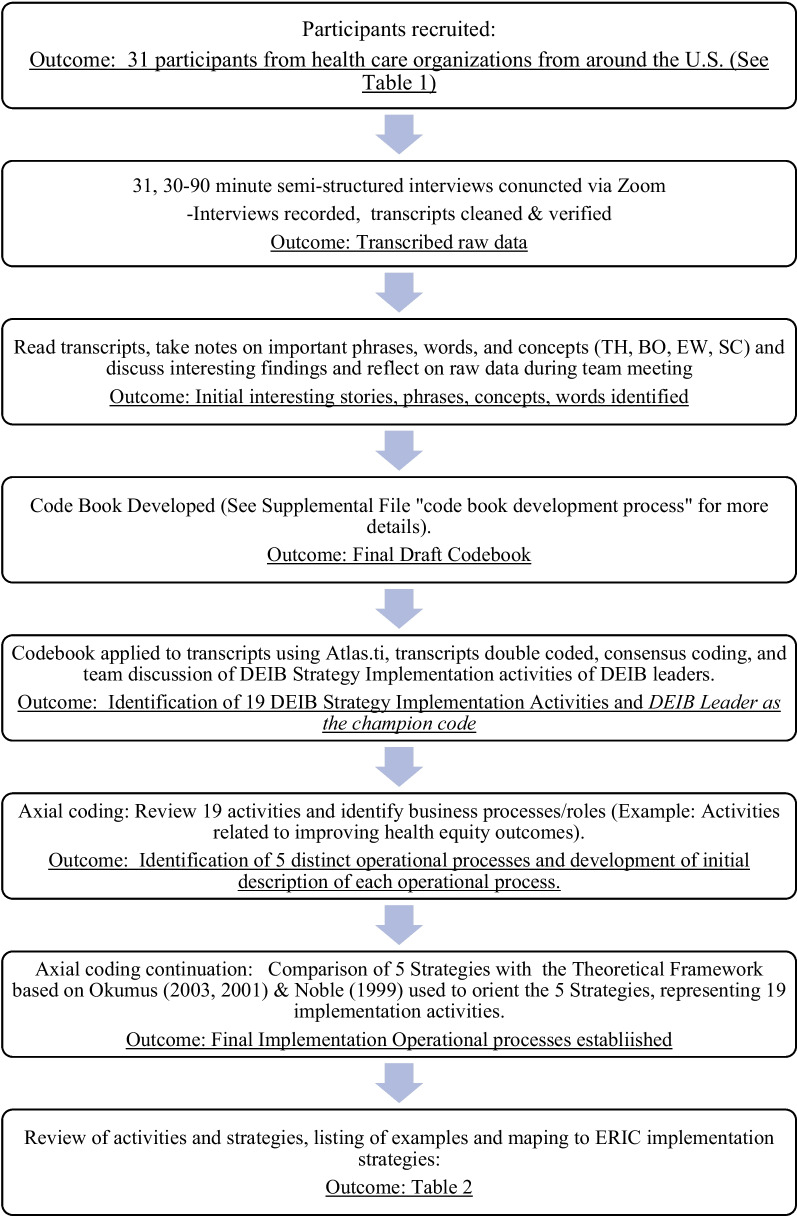
Analytical approach

We analyzed the transcripts using a hybrid inductive-deductive approach to identify DEIB top-level leader roles and the process of DEIB strategy implementation. Inductive analysis was used during the initial coding and deductive logic was used during axial coding to examine and understand the findings through the context of our theoretical framework. The following section describes the development of the codebook, the coding process, and the steps taken to contextualize our findings within our theoretical framework which is grounded in a strategic implementation framework [25, 26]. A visualization of the steps which are described during this section is provided in Fig. 1.

The team used a multi-step approach to develop the codebook which is based on MacQueen et al. [29]. Codebooks were iteratively tested independently by TH, BO, EW, and SC and modified upon a discussion of the testing outcome until the group established there were no further revisions to the codebook. For demographic characteristics of participants, categories were created using the respondent’s own terms, which were then grouped together by the research team where appropriate. The study team adopted this approach because we felt it respected the input from our study participants whose perspectives are under-represented within in HCO research. We report groups which were present in our study population; therefore, any demographic groups not listed in the table indicate that we did not have any participants self-identify in such groups. A more detailed description and visualization of the entire coding process is available in Additional file 3 (“code book development figure”). The completion of interviews and subsequent coding continued until saturation was met, which occurred as the collection of additional data did not provoke any new theoretical development.

Following the development of the codebook, the research team (TH, BO, EW, and SC) used NVivo to code the interview transcripts, with each transcript being double-coded. The team met during this time and addressed any questions or concerns that arose during the coding process. Upon completion of the coding assignments, team members met with the other person who had coded each transcript and compared coding statements across each coded transcript. Disagreements or differences were discussed until a consensus was met. This approach was adopted due to the complex and exploratory nature of the study. The process of double-coding each interview transcript enabled the team to increase coding validity (using multiple coders), while also ensuring reliability and credibility (consensus coding) [30, 31]. This resulted in the identification of the *DEIB leader as the champion* as well as 19 DEIB activities that the top-level leader was responsible for.

Next, we sought to understand how the 19 DEIB strategy implementation activities may fit within the broader process of strategy implementation, so we conducted an iterative analytical process, utilizing axial coding to examine the relationship between our 19 activities. This part of the analysis began by independently grouping each activity together with other activities that described business processes that seemed to be oriented in similar concepts or areas of the business. For example, during this time, we identified that there were a set of implementation activities which represented a broader strategy that involved how DEIB leaders have responsibilities and roles related to addressing or improving health equity outcomes. As a result of this, we identified 5 DEIB implementation strategies. Next, we wrote an initial description of each grouping. Following this, to contextualize our findings about DEIB leader work within the broader institutional strategy implementation process, we compared our identified groupings with our theoretical framework, spending the most significant time comparing the 5 *operational process* variables and definitions identified in Okumus’ [25, 26] *Strategic Implementation Framework* (Operational Planning, Resource Allocation, Communication, People, Monitoring and Feedback, and External Partners) with the groupings we identified. Following this comparison, we conducted an iterative process that included comparing Okumus’ definitions with the descriptions and groupings we identified. When our groupings and descriptions were similar, we adopted Okumus’ the operational process label/name for each grouping. When our grouping’s descriptions did not align with Okumus’ operational process label/name, we maintained our descriptions and definitions.

## Results

Information about participant’s self-described characteristics and backgrounds is displayed in Table 1. The categories for these demographics use the participant’s own terms from the interview—as such, every term in the table comes directly from study respondents’ direct response to question 1 of the interview guide. Interviewees were mostly Black, majority female, and mostly heterosexual and had a variety of degree backgrounds and direct supervisors.

**Table 1:** Self reported identities and backgrounds of participants

Characteristic	% of recorded respondents
**Gender**	
Female/woman	59%
Male/man	41%
**Race**	
Black/African American	70%
White	7%
Latino/a	7%
Asian	7%
Multiracial	7%
**Sexual orientation**	
Heterosexual/straight	88%
Homosexual/gay	8%
Other	4%
**Degree type**	
Business (MBA, MHA, MHRM, PhD)	42%
Other non-PhD (MPH, MSW, etc.)	23%
Non-Business PhD	19%
MD	12%
JD	4%
**Direct supervisor**	
Chief Executive Officer/President	48%
Chief Human Resources Officer/Chief Administrative Officer	19%
Not applicable—Consultant Role	7%
Chief Diversity Officer	7%
Vice President of Community Health	7%
Chief Physician Officer	4%
Vice President of Population Health	4%
Vice President of Strategy	4%

The main finding of our study is the operationalization of how organizations are implementing DEIB interventions and identifying the role of the DEIB leader within the context of DEIB interventions. We identified context-specific implementation strategies which are unique to the implementation of DEIB organizational interventions compared to traditional organizational interventions related to organizational change.

We identified the DEIB leader as the champion of the implementation strategies and identified five strategies which describe the role of DEIB top-level leaders within DEIB intervention implementation: (1) *People*, (2) *Health Equity*, (3) *Monitoring and Feedback*, (4) *Operational Planning and Communication*, and (5) *External Partners*. Of these five, *People*, *Monitoring and Feedback*, and *External Partners* aligned with the theoretical framework we used to frame and understand the DEIB organizational interventions, top-level DEIB leaders, and DEIB implementation strategies. *Health Equity*, *Operational Planning*, *and Communication* were found to be specific and unique compared to the original framework where Okumus described how organizations implement interventions. These unique strategies explain the complexities of DEIB interventions and implementation strategies as compared to traditional implementation strategies adopted by organizations. Within these processes, we identified 19 significant initiatives for which DEIB leaders were responsible within an implementation strategy in this space, which have previously never been identified within the field of strategy implementation, implementation science, or studies examining the roles of DEIB top-level leaders. Our findings synthesize the operational processes that were present throughout the sample, but not necessarily in every healthcare organization’s DEIB implementation process. The results of our findings are described in detail using illustrative quotes, within the body of this section of the manuscript. Next, we also present these findings in Table 2 where we provide examples of each strategy, a mapping of the DEIB strategies to the ERIC discrete implementation strategies, and identify relevant literature related to each activity [32]. We mapped the DEIB strategies to the ERIC discrete implementation strategies to support the consistent classification of strategies throughout implementation science as well as to ensure readers understood the mechanisms adopted by DEIB top-level leaders within the context of implementation science.

**Table 2:** Implementation strategies, examples, and relevant literature

DEIB implementation strategies	Examples identified by participants	Mapping to ERIC implementation strategies	Relevant literature
*People*			
Talent recruitment, and retention:	- Tailor recruitment and retention policies and incorporate inclusive practices to these policies - Provide technical assistance and consultation to writing job advertisement and job descriptions to ensure language is inclusive - Change guidelines to include equitable search processes - Develop a formal implementation blueprint for how the organization will embody inclusive strategic human resource management	- Provide ongoing consultation, conduct educational meetings - Develop a formal implementation blueprint - Recruit, designate, and train for leadership	[33–35]
Employee learning:	- Develop and distribute corporate learning materials to support DEIB interventions to train and educate stakeholders - Integrate DEIB-related training into the existing corporate learning system - Develop incentives to encourage DEIB corporate learning within the organization	*-* Distribute educational materials - Alter incentive/allowance structures	[36–40]
Employee resource groups:	- Formalize ERGs within organization to support DEIB dissemination - Allocating budget to ERGs - Adopting ERG representation within corporate governance to serve as champions	- Use advisory workgroup - Utilize financial strategies - Identify and prepare champions	[41–43]
Formal workforce development and talent pipeline	- Develope resources that allow employees from minoritized populations to develop skills that will enhance their future job growth and enter leadership roles - Lead leadership training programs targeted at employees who are part of minoritized populations and/or traditionally under-represented within leadership - Adopt mentoring and coaching programs for employees from minoritized populations to identify	*-* Distribute educational materials - Conduct educational meetings - Recruit, designate, and train for leadership	[44–47]
Engaging top-level leaders:	- Promoting importance of the DEIB interventions to other health system leaders, such as the Board of Trustees and CEO through formal and informal channels - Developing one-on-one relationships with board members to share knowledge and expertise related to DEIB topics and gain support	- Facilitation - Obtain formal commitments - Involve executive board - Build a coalition	[48]
*Health Equity*			
Reduce disparities in clinical outcomes, access, and patient experience	- Establish a committee of clinical leaders to identify barriers minoritized populations experience when equitable health care in their organizations - Inform clinical leaders of expectations regarding the incorporation of health equity into clinical care - Establish a regular operating routine for reviewing health disparities and health equity data from their organization - Identify and document what mid-level leaders and frontline staff are already doing to address disparities in clinical outcomes and access and establish future goals for these initiatives	- Build a coalition - Inform local opinion leaders - Identify early adopters - Capture and share local knowledge - Change records systems	[49–51]
Health equity research	- Creating a research center for Health Equity with a mission to support research focused on alleviating healthcare inequities - Creating systems and providing research support for clinicians engaging in health equity research - Identifying research agenda for the organization that advances the DEIB interventions	- Build a coalition - Capture and share local knowledge	[4,51
Language translation services	- Evaluating outcomes and experience utilizing existing translational services and identifying opportunities for growth - Incorporating translation services outcome metric into organization scorecard - Conduct a needs assessment to identify opportunities for creating patient-related materials (handouts, signage, patient portals) that are easily understood and use inclusive language - Ensuring that health system translation services are equitable and accessible to all patients - Managing translational services staff	- Audit and provide feedback - Change record systems - Facilitation - Conducting local needs assessment	[52, 53]
Community health outcomes evaluation	- Identifying metrics to monitor population health outcomes within underserved communities or communities with large minoritized populations - Incorporating community health metrics into the organizational performance evaluation - Reporting community health outcomes of under-served populations or minoritized populations	- Develop and implement tools for quality monitoring - Change record systems - Audit and provide feedback	[54]
*Monitoring and Feedback*			
Board engagement	- Presenting at board meetings on key DEIB priorities and/or initiatives within the organization - Establishing relationships with board members and board sub-committees to support the engagement of boards of DEIB issues - Serving as DEIB subject matter expert to board	- Facilitation - Promote adaptability - Involve executive board - obtain formal commitments	[55]
DEIB scorecards	- Developing key metrics that provide evidence of DEIB implementation success in the organization - Establish sources of data and reporting routine to regularly obtain data on health equity outcomes on time - Mapping data within systems to support scorecards	- Purposefully reexamine the implementation - Develop and organize quality monitoring system	[56]
Listening sessions	- Events with organization leaders and employees present to support opportunities to voice thoughts about organizational culture and areas for improvement - Engage in listening sessions after critical events	- Conduct local needs assessments - Obtain and use patients/consumers/and family feedback	[57]
*Operational Planning and Communicating*			
Negotiating the DEIB leader role	- Working with other organization leaders to demarcate specific responsibilities of DEIB position and determine workplan for areas of overlap between roles - Negotiate with other top-level leaders on how to partner on implementation strategies which require collaboration	- Facilitation - Inform local opinion leaders	[58]
Building collaboration to support sustainable change	- Meeting with employees across the organizational hierarchy to support buy-in - Developing buy-in with top-level leadership team	- Build a coalition - Conduct local consensus discussions	[59, 60]
Developing DEIB personnel infrastructure	- Hire and train DEIB leader direct reports - Establish staffing needs to support DEIB implementation strategies - Gain access to funding to hire DEIB staff through organization’s budget process	- Centralize technical assistance - Develop a formal implementation blueprint - Access new funding	[61]
Developing governance structure	- Building a framework for the implementation and maintenance of DEIB interventions - Establish DEIB leadership committee and document guidelines for committee - Building routines/processes and organizing meetings that support effective decision-making of DEIB issues	- Use Advisory boards and work groups - Provide interactive assistance	[41, 62, 63]
DEIB consultation to support DEIB strategies	- Meeting with organization leaders to support other leaders’ specific DEIB priorities or initiatives - Engage in tacit knowledge to support change management processes - Serve as a consultant and subject matter expert to support systematic integration of DEIB into all levels of organization - DEIB top-level leader establishes themselves as the DEIB resource and subject matter expert	- Provide local technical assistance - Facilitation - Inform local opinion leaders - Centralize technical assistance	[64, 65]
*External Engagement*			
Community engagement	- Meeting with community leaders to assess social needs and creating a plan to alleviate them - Collaborate with the organization’s existing community engagement team to identify opportunities to improve engagement with communities with large populations of minoritized populations	- Build a coalition - Conduct local consensus discussion	[54]
Supplier diversity	- Sourcing hair and skin care that meets the needs of minorized populations - Collaborating with finance/purchasing team to diversify organization’s suppliers - Establishing sourcing targets related to engaging with a diverse group of 3^rd^ party partners	- Mandate change - Change physical structure and equipment	[66, 67]

### DEIB leader as the champion

DEIB Leaders stated that their primary responsibility within the organization was managing and driving all DEIB strategy efforts throughout the organization. They described that they were often hired by their healthcare delivery organization’s board or CEO to lead DEIB work in the organization. For example, one participant stated, “I lead the shaping of our organization as one that values diversity, equity, and inclusion…working around and leading our strategic development, promotion and implementation of a variety of initiatives.” (Participant #10).

### DEIB implementation strategies led by the DEIB leader

#### People

This strategy describes a set of DEIB leader responsibilities that relate to a coherent approach to the implementation of DEIB initiatives within the existing human resource management systems of a healthcare delivery organization, as well as aligning the existing human resources systems to support an inclusive and equitable culture within the organization. DEIB leaders described that broadly speaking, a significant component of their position is focused on creating an environment within the organization that is inclusive and enables all individuals to thrive. It was often described that equity must start within the organization as it pertains to how the organization supports its employees. There are four activities within this implementation strategy:

##### Talent recruitment and retention

DEIB leaders are developing and implementing hiring and retention policies to ensure equitable hiring, recruitment, and retention practices. DEIB leaders described working in partnership with the organization’s existing talent acquisition team to implement human resources policies that align with and facilitate the DEIB strategy.

From a talent acquisition perspective, we want to make sure we have the broadest reach that we possibly can we're casting our net we're focusing on recruiting within our communities, so that the people that we recruit look like the communities that we serve. (Participant #7)

DEIB leaders may also serve as advisors or provide subject matter expertise during the development of employee learning materials (courses, training sessions) that address underlying inequities in talent recruitment and retention practices among organizations.

##### Employee learning

DEIB leaders oversee the identification of the learning needs of the organization that best support the workforce development germane to achieving the strategic plan. Some participants described working closely with the existing employee learning training infrastructure or team to achieve this.

So, for instance, last year, part of one of our balanced scorecard priorities was providing bias awareness training to all colleagues, so my team in conjunction with our learning and organizational development team select the content and then came up with the method through which we were going to distribute that to people across the system. (Participant #4)

These leaders may also be solely responsible (within their team or themselves) for developing the content and conducting employee trainings. Employee learning opportunities were being used within the DEIB strategy to address the cultural norms within the organization, (e.g., cross-cultural communication) as well as to promote fair and equitable processes within the organization (e.g., implicit bias training).

##### Employee resource groups

The implementation of organizational DEIB interventions includes the development and support of employee resource groups, which are defined as committees of an organization’s employees who share a common identity and work towards creating a more inclusive work environment. DEIB leaders describe they are responsible for driving the broader utilization of employee research groups within the DEIB strategic context of the organization, alongside overseeing their development, daily functioning, and budget.

I am someone now who can help with that and create a strategic plan for those employee resource groups that is tied to our strategic, [organization’s name] overall strategic plan, so they’re not just clubs hanging around hanging out going to happy hour, they have the business case and business initiative, that are connected to what we do as a system. (Participant #15)

##### Formal workforce development and talent pipeline

This describes how HCOs developed formalized approaches to recruit and train talent within their organization. DEIB organizational interventions support the development of diverse talent pipelines, which are an important avenue for both new and established employees to build competencies to ensure their skillsets align with future job growth. DEIB leaders oversee the development and transformation of existing leadership development programs, or they are creating talent development programs which are inclusive of individuals from minoritized populations, with the goal of building a foundation to support the organization’s future labor force needs.

We have been able to identify and develop high potential employees, so we are creating a talent pipeline, so trying to figure out like, we have a CEO development program that we want to diversify. And because that we’ve identified our key hospital management positions as those having an opportunity to have more diversity. (Participant #13)

##### Engaging top-level leaders

This describes how DEIB leaders work to support the development and growth of other top-level leaders in their organization to be able to communicate and engage with the DEIB strategy and issues in the organization.

So, I learned my first year that our leaders were not comfortable. So, this year we’re focused on leadership development around these topics because they’re not comfortable talking about culture, talking about race and ethnicity, talking about inclusion or any of those areas. I mean I'm not saying all leaders, but we got quite a few that are not... (Participant #23)

DEIB leaders describe that to be successful in this activity they work to develop relationships with their peers to build trust. Other top-level leaders may also approach the DEIB leader to coach them on issues related to DEIB.

#### Health equity

This operational process describes activities of DEIB top-level leaders that aim to advance the organization’s ability to address health equity and disparities in access, outcomes, treatment, and quality of care experienced by patients. A significant component of their role is to align the patient experience with the organization’s mission, vision, and values, which includes health equity and diversity dimensions. These processes and strategies are unique and exclusive enough to DEIB as it warrants the expansion of the original theoretical framework. There are four activities within the health equity operational processes:

##### Reduce disparities in clinical outcomes, access, and patient experience

DEIB leaders work to identify a systemic approach to reduce disparities in access, quality, and healthcare outcomes. Leaders described that their organization’s DEIB vision prompted them to partner closely with the existing patient experience, quality, and safety, medical, and nursing leadership to develop a clear understanding of the current initiatives, the areas of improvement, approach to monitoring outcomes, access, disparities, and patient experience of patients who are from minoritized communities.

kind of like what the DEI looks like for our patients and families, and then even our community so like, you know, a large scope of my job right now is really thinking about health equity, you know what would that look like for [organization name removed], how do we think about, you know, launching or at least, creating an inventory of all the health equity work across the hospital (Participant #22)

DEIB leaders also described that while there is evidence that disparities exist, the next steps the organization should be taking to reduce disparities are less clear. As a result, DEIB leaders facilitated work groups or committees to identify the outcomes the organization wanted to focus on.

##### Health equity research

DEIB leaders are developing, strategizing, and building research centers that focus primarily on health equity and advancing the contribution their organization makes to the scholarship in this area.

And I envisioned that the Center for Health Equity would become the research and evaluation arm of this work. Given that we have talent all across campus, my hope is to be able to tap into that talent and interest, including you, obviously, so that we can begin to create a vibrant phase of work of scholarship and in the broad areas of health equity across social and economic, psychological, and physical health domains. (Participant #24)

##### Language translation services

DEIB leaders are often responsible for the language translation services department and its associated employees. Language translation services describe a process where English translation is provided to patients and family members who do not speak English. DEIB leaders sometimes managed employees within this critical service for patient accessibility and communication, as well as any contracts the organization had as a part of providing translation services.

I am fully responsible for language services across the system, so interpretation and translation. (Participant #19)

##### Community health outcomes evaluation

DEIB leaders facilitate and oversee a systematic approach for the organization to measure and evaluate the health of the community as it relates to a broad set of health, social, economic, and environmental disparities.

We do robust measurement of the community health, I’ll call it the dashboard that we use that we ourselves developed in partnership with a with a nonprofit organization, is all about underpinnings of equity and health related disparities, so not just medical care disparities, but more particularly social and economic disparities, health behavior disparities, physical environment disparities, as well as disparities and clinical care which is primarily… Access and quality related based on social and economic factors to include race, ethnicity, sexual orientation, gender, all those things, right. (Participant #8)

Such evaluations served to support a broader and more contextualized understanding of the populations served by the organization, as well as to facilitate future DEIB interventions.

#### Monitoring and feedback

This describes the activities that DEIB leaders do to facilitate informal and formal ways of monitoring activities being carried out during the implementation process.

##### Board engagement

DEIB leaders frequently present and meet with the organization’s board regarding the status of DEIB strategic plan implementation. This may include seeking their approval, presenting the status of various projects, and requesting input for changes or variations from the original DEIB strategic plan.

Sure, I have presented both to our system level boards and we also have hospital advisory boards that I’ve done some presenting with as well. And so far, because of the timing that has happened virtually, I look forward to the opportunity now that we can start kind of having those meetings in person to be able to do so in person. And so you know I think it’s I was a good experience that I was invited back (Participant #10)

DEIB leaders also expressed that boards are intimately involved and responsible for the implementation of the DEIB strategies/interventions. This type of involvement has supported the DEIB leaders’ ability to drive and lead the execution of the DEIB interventions, as it communicates the importance of the initiatives to other decision-makers in the organization. It also reinforces values, holds other leaders accountable, and can allocate resources to support the work.

##### DEIB scorecards

DEIB leaders are responsible for identifying and developing items for an already existing scorecard, or for a new scorecard. Scorecards with DEIB measurements help ensure that all leaders within the organization (board, top-level, committees) are aware of the accomplishments and opportunities the organization has with regard to the implementation of the strategy.

So, we have a balanced scorecard that we use to govern our entire organization. In one of the quadrants is the culture quadrant, and so our DE&I and culture data is input into that quadrant. And that is reported out on a quarterly basis, and then on an annual basis. And the metrics in that quadrant drive compensation. (Participant #12)

##### Listening sessions

Some DEIB leaders lead and facilitate listening sessions between top-level leaders and other employees. These may occur following a major event, such as the murder of George Floyd or Breonna Taylor. The DEIB leaders monitor the external environment and the experience of employees within the organization as well as organizing and facilitating listening sessions when needed.

We did [removed] listening sessions… And it was really leaders that had to get out of their comfort zone because they didn’t know me, and so I was a complete stranger telling them that they had to do something that they’ve never done and do it in a very vulnerable, authentic way because it was a real moment right and so not to oversell what we were going to do, but we accelerated what we had to do because out of those listening sessions. (Participant #18)

#### Operational planning and communicating

This describes the development of infrastructure, processes, communication channels, and interpersonal relationships that support the implementation of DEIB initiatives. Much of the DEIB leader’s work is predicated on the concept that the organization may need to change processes or policies, or human behaviors that have existed in the organizations for decades. Five activities describe this process:

##### Negotiating the DEIB leader role

DEIB leaders are constantly working through formal and informal channels to determine and establish what the DEIB top-level leader function should include and how to best position the role in the organization to achieve the organizational strategies. This may include approaching specific opportunities as a trial and error, directly negotiating or making a case to their supervisor for function autonomy and brainstorming with other top-level leaders to determine the best strategies to support the organizational DEIB interventions. Such work is laborious and is related to the evolving scope of DEIB values and strategies in organizations and our society. DEIB leaders were establishing what parts of the work should be completed by their team as opposed to holding another function responsible.

I think my responsibility is to help identify ways to support and amplify the work that’s working that’s going well, and also to measure what’s working well, and to be able to be really transparent about where we need help by looking at the numbers of like retention and things and courses. So but I don’t feel like I’m in it by myself. I feel like everybody is kind of doing stuff, and but they what they really could use is some support in terms of knowing what other people are doing and coordination across all of these different elements (Participant #29)

##### Building collaborations to support sustainable change

DEIB leaders lead change in their organization by building relationships across the organization. This created trust and buy-in from other leaders, which facilitated the furthering of the DEIB vision.

I would say I wouldn’t have any success if I didn’t have the relationships that I have... and when I mean collaboration, I really want folks to understand that this is critical to not just my role, but also the roles that they are individually serving as well (Participant #17)

##### Developing DEIB personnel infrastructure

DEIB leaders are building and advocating for the team and personnel needed to support the business systems and organizational change associated with implementing the DEIB strategies/interventions. A central component of achieving organizational change that emblemizes DEIB is the existence of an established team and budget. However, many leaders were constantly reckoning with a lack of resources, which required substantial effort put towards creating a sustainable infrastructure:

So, it’s just a matter of how you organize it, but I would say unequivocally we’re not adequately resourced now based on the breadth of what we want to do, but we do have some open positions that we’re trying to get filled. (Participant #8)

##### Developing governance structures

DEIB leaders are building infrastructure to guide decision-making within the organization to support DEIB organizational interventions. Each organization has an existing governance structure, which refers to the rules, procedures, roles, and division of responsibilities within a decision-making process and most notably defines the role of the board and executives in decision-making. DEIB leaders work with different stakeholders within the governance structure (e.g., Board, CEO, Committees, Councils) to establish and manage expectations for how these groups will support the DEIB organizational interventions as well as potentially establish and negotiate for new formalized roles and processes when needed.

I’ve recruited a number of individuals to create a governance structure … and so we created a governance of diversity and equity committee for them, share the framework, um, walking hand in hand with them in terms of setting goals for their campus that are aligned with the overarching goals of the equity plan at the organization (Participant #21)

##### DEIB consultation to support DEIB interventions

DEIB leaders communicate the DEIB initiative implementation plan and encourage implementation through informal communication channels, which frequently involves consultation with other executive function leaders so they can drive implementation of the DEIB strategy.

I’m a part of a huge organization and there’s only two of us, so I can’t lead everything, I can't be a part of everything. So, that’s why I really kind of want to act as a consultant to some of these leaders to help get them up to speed (Participant #13)

#### External partners

DEIB leaders support the development of processes or policies to support the ways the health delivery system engages with other organizations or community members. Within this, there are two activities: community engagement and supplier diversity.

##### Community engagement

This is defined as the DEIB leaders working to understand current practices related to community engagement and then identifying ways to improve engagement with people and communities who in the past have been excluded and disenfranchised. To do this, DEIB leaders work alongside the organization’s community engagement team, who are responsible for the application of institutional resources to address issues facing communities they provide healthcare services for. DEIB leaders are responsible for understanding the current practices of these teams and establishing ways that community engagement can be done to support communities which have been excluded or disenfranchised. They also partner with the existing community engagement teams to identify ways that their team can support the DEIB organizational interventions.

I help to inform a strategy, support the synergies that are necessary because what we find is that the community wants certain things, and the hospital designs things in a different way. So, how do we make those much more closely aligned, so that there is more impact for the limited resources that we actually have, both the community in organization. (Participant #21)

##### Supplier diversity

DEIB leaders partner with the finance and/or purchasing team to implement a supplier strategy that incorporates the sourcing of products, which support their DEIB strategies/interventions, and to grow the percentage of supplier spending among minority-owned businesses or contracts.

The supplier person has kind of a dotted line to me and I consult a lot on the way they’re collecting those kinds of data and then how they’re thinking about a long-term plan for some of our suppliers. (Participant #30)

## Discussion

Our study adds to implementation science by providing a significant contribution to identifying *how* DEIB top-level leaders may impact the implementation of DEIB interventions within HCOs. We defined DEIB leaders as the champions and identified implementation strategies and activities which may occur when implementing DEIB interventions in HCOs. Our work demonstrates the relevance of Okumus’ *Strategy Implementation Framework* in the context of DEIB interventions at HCOs and builds out both implementation strategies and specific activities that DEIB leaders are responsible for as the champions of these initiatives. The opportunity presented by the emergence of a new strategic focus across organizations on DEIB strategies/interventions, in combination with the emergence of new DEIB top-level leadership roles created a unique opportunity to understand how top-level leaders contribute to the development and achievement of organizational-wide interventions. To date, much of the research on DEIB initiatives in HCOs has focused on what interventions should be adopted [68–70]. This study is the first to provide an empirically developed, list of implementation strategies and activities that encompass the implementation strategies of organizational DEIB interventions led by the top-level. Implementation has long been a challenge for organizations and our framework can be utilized by researchers and healthcare delivery system leaders (Boards, CEOs, c-Suite leaders, administrators) to evaluate effectiveness, understand and design the implementation of DEIB strategies/interventions, allocate organizational resources, define the role of the top-level leaders within the process, as well as troubleshoot and improve their current processes associated with adopting DEIB strategies/interventions. In light of the widespread adoption of DEIB leaders, we do not yet understand the combined effectiveness of systematically implementing these strategies. We were able to identify supporting evidence for many of the 19 activities (see Table 2), and our results highlight relevant support across some organizational settings. There are significant opportunities to understand how these activities work collectively to support HCOs.

Additionally, our findings provide valuable insights into the inner workings of how healthcare organizations are driving DEIB change, which can be used by policymakers to better identify ways to incentivize and support the work to improve health equity by delivery organizations throughout the entire healthcare delivery system. While we were able to identify consistent implementation activities in our study, there is significant variability in the different adoption activities across participants, and subsequently healthcare delivery organizations. Policymakers could utilize payment programs and funding mechanisms to incentivize the widescale adoption of HCO DEIB strategies and interventions.

Our findings also have implications for the concept of leadership in implementation science. Our study builds on the clear evidence that top-level leaders are vital to the implementation of evidence-based interventions, by defining the specific activities top-level leaders do, which support organizational change, as opposed to the change of individuals within an organization. Identifying the DEIB top-level leader as the champion within the implementation process contributes to the advancement of how top-level leaders support implementation and is in line with previous research which states that implementation relies on leaders in designated roles who have the responsibility of facilitating the implementation process. Our study further defines this concept and specifies how leaders act as champions. The results presented here demonstrate that in addition to leadership style, the way leaders develop processes, execute on broad concepts, and operationalize organizational-wide change is important to consider when trying to understand evidence-based intervention sustainment and implementation.

We also mapped our findings to the ERIC implementation strategies to support the use of consistent terminology within the field of implementation science and the applicability of our findings to understanding how organizations choose strategies to support interventions aimed at changing the organization. While we were able to identify and map many of the DEIB strategies within ERIC strategies, the mapping was challenging at times. There are two possible explanations; first, it may be because DEIB interventions are unique and especially challenging for organizations to implement; therefore, they are adopting innovative and unique strategies which may not be currently documented with the ERIC strategies. Another explanation could be that this study examined organizational-level interventions and the ERIC strategies do not specify which level within an organization they operate. Some ERIC strategy descriptions infer that they may take place or be appropriate at individual/intraorganizational/interorganizational level, but the clear delineation of these concepts is not articulated. Future research in this area which focuses on the classification of strategies by the level of the organization strategies (micro, macro, and meso) occur could support a more consistent application of strategies when examining the implementation and adoption of organizational-level interventions. Consistent with previous studies of the role which were not as methodologically rigorous, we identified that DEIB leaders have responsibilities that are “broad-spanning” [6, 33]. Despite the consensus that DEIB leaders oversee a broad spanning set of responsibilities, our study did not identify DEIB leaders as having significant responsibilities regarding the allocation of resources to support DEIB strategic initiatives. Resource allocation, management, and alignment are major components of effective implementation [25, 26]. This finding is concerning as we anticipate that DEIB strategies/interventions may require significant resources to accomplish. The study of leaders (top, middle, front-line) often focuses on how these leaders can support a climate which supports implementation activities. Further research is needed to understand how organizations and top-level leaders allocate resources to support the implementation strategies for DEIB organizational interventions.

Relatedly, while the DEIB leader may serve as *champion* of implementing these initiatives, the overarching organizational commitment to DEIB spanning across the entire leadership team is an important consideration as well. The difference between DEIB interventions as a siloed project within the top-level and an institutional imperative may come down to this broader degree of commitment, a topic ripe for future research that builds off our current results. Additional future work will also focus on the relationship between the DEIB leader’s role as the champion of DEIB initiatives and the overall organizational mission- in other words, examining how DEIB implementation strategies act as mechanisms to drive broader organizational change or are inhibited by inertia and/or resistance from others who are not bought in.

### Limitations

Despite the valuable contributions of our research, our study has limitations which should be noted. First, our recruitment strategy relied heavily on a snowballing approach; this may have resulted in a particular participant sample. Additionally, our study could also be enriched by gaining additional perspectives (e.g., board members, CEO, C-suite leaders, clinical leaders who work with the DEIB leaders, etc.). Such groups were described by participants as being a part of the implementation strategies of DEIB organizational interventions. These additional perspectives may be able to support a richer description. Despite these limitations, we believe this study adds to the limited knowledge about DEIB leaders and the implementation of DEIB strategic initiatives by healthcare delivery organizations.

## Conclusion

Many HCOs have made public statements declaring that they are committed to addressing health inequities and improving their workplace to all employees can be successful and contribute. To move beyond words, and bring the type of change that is needed, the organization must focus on not only establishing a strategic plan that embodies DEIB strategies and interventions, but also implementing them. The identification of the DEIB leader within this process advances our understanding of how organizations are working to change. As history suggests, changing human behavior, and addressing systematic racism within organizations is hard work, it does not happen fast and will require organizations to troubleshoot along the way. Our contextualization of the DEIB leadership positions provides an understanding of how such organizations are incorporating top-level leaders to address these sorts of challenges. Our work can help HCOs systematically identify the individuals and functions of the organization that must be at the table to drive DEIB change.

### Supplementary Information

**Additional file 1. **ISSM COREQ Checklist.

**Additional file 2. **Interview guide.

**Additional file 3. **Code book development figure.

## Data Availability

No datasets are available from this study owing to the consent given by participants, which limits data to the research team only.

## Abbreviations

AMC: Academic Medical Center
CEO: Chief Executive Officer
DEIB: Diversity, Equity, Inclusion and Belonging
IRB: Institutional Review Board
HCO: Health Care Organizations
