# Supplementary material for: Top-level leaders and implementation strategies to support organizational diversity, equity, inclusion, and belonging (DEIB) interventions: a qualitative study of top-level DEIB leaders in healthcare organizations
Source: Implement Sci. 2023 Nov 7;18:59. doi: 10.1186/s13012-023-01319-7 (PMC10631201; doi:10.1186/s13012-023-01319-7)
Supplement: Supplementary file 2 — Additional file 2. Interview guide. [file 13012_2023_1319_MOESM2_ESM.docx]

**Supplemental Material, Additional file 2: Interview guide**

Sections

1. ***We are going to ask you some information to understand your Personal Background and how you identify (identity info):***

- How do you gender identify? -
- How do you identify racially?
- How do you identify culturally/ethnically?
- What is your sexual orientation/identity?
- What is your training/degrees/professional background? (Degree?)
- Before working in a DEI role, what was you primary function (eg – nursing, HR, Ops, clinician (MD/DO), marketing, communications, sales)

1. ***Thanks, Now we want to learn about what you do as a part of your role.***
   1. Can you tell me your job title, how long you have been in this role, and what you do as a part of your role.
      1. Prior to this role, have you served in another DEI leadership position? If so, for how long

*Follow-up/prompts:*

Do you oversee any of these? What do you do when you oversee?

If not, who is responsible for these functions?

- Health equity – Clinical outcomes/access/disparities
- Community engagement
- Supplier Diversity
- Strategic planning and DEI initiatives
- Employee DEI training/corporate learning
- Workforce diversity
  - Recruiting/retention
  - Affinity groups (

Which of these do you performance goals related to each of these?

1. **Next I would like to understand your reporting structure, Who do you report to? (And who does that person report to)**
   1. Follow-up:
      1. How would you describe your relationship with your direct supervisor? What about with other C-suite team members? How would you describe the importance of these relationships with your success in achieving success in your role?
      2. Do you ever present/meet with the board? How would you describe your relationship with them? How would you describe their involvement in DEI initiatives?
      3. How many people do you have reporting to you? What are their titles? What is your budget like in a year?

*try to figure out the org structure/relation to C-suite.

1. **Next, I would like to talk about your organizations DEI goals and initiatives, What are your organization’s institutional goals for DEI and what role do you play in these?**

**Follow-up:**

- 1. How was this goal established?
  2. What was your role in the process?
  3. What is your role in achieving these goals? Do you have metrics for these? How are these measured?

1. **Professional Background**
   1. How does your “personal identity” influence /contribute to your job experience, your role and your professional success as a DEI leader?
   2. There is a high turn over among CDO/DEIE leaders , why do you think that is?
   3. To what degree do you feel your previous professional experiences contribute to your current role?
   4. What resources do you use to develop your capabilities in your current role What would you say are the essential knowledge, skills, and abilities for someone who would be interested in serving in a similar role?
   5. To what degree do you think your previous training or professional experience prepared you for this position? Did you ever seek additional training as a part of this role? (eg – MBA, Certificate, training in any way)

**FINAL QUESTIONS**

1. What do you think are the most important things that need to occur for a CDO/CEIO, (etc) to be successful?
2. Is there anything that you think has made you successful that we haven’t talked about? Or any barriers you want me to understand?

- What major changes have you observed in the last couple of years in your career? Where do you see it going? What do you project how this will evolve?
