# Supplementary material for: Top-level leaders and implementation strategies to support organizational diversity, equity, inclusion, and belonging (DEIB) interventions: a qualitative study of top-level DEIB leaders in healthcare organizations
Source: Implement Sci. 2023 Nov 7;18:59. doi: 10.1186/s13012-023-01319-7 (PMC10631201; doi:10.1186/s13012-023-01319-7)

Supplemental Material, Additional file 3: Code Book Development Figure

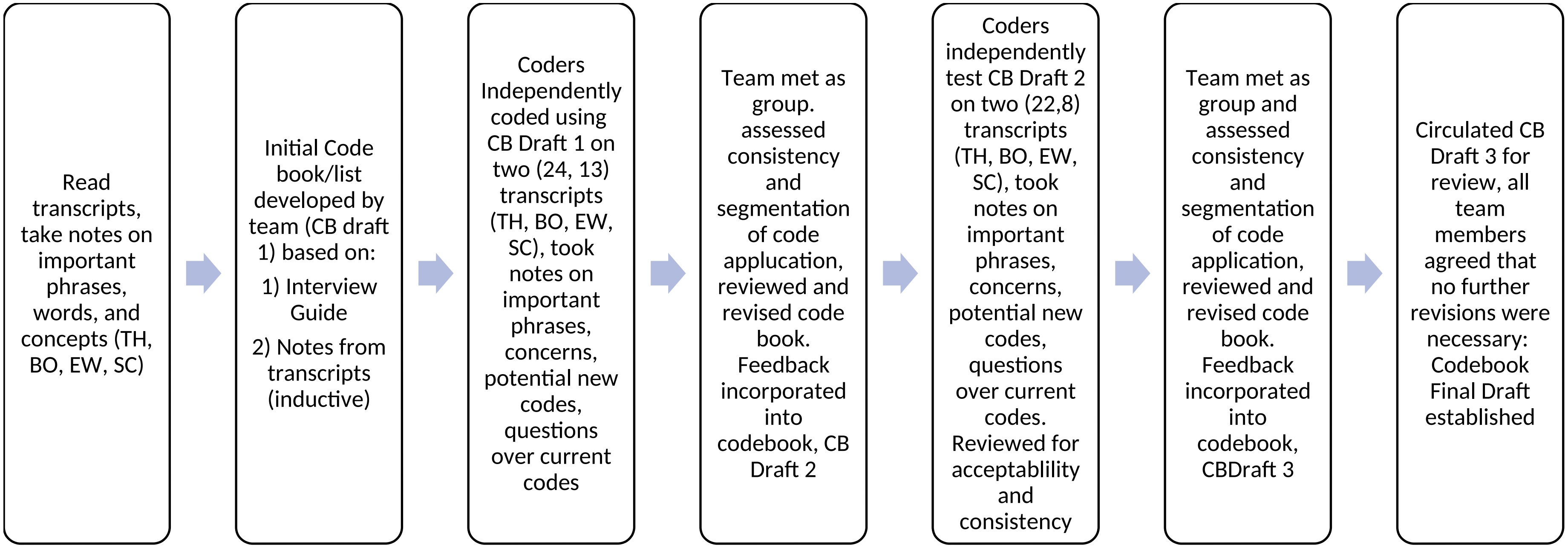

Supplement: Supplementary file 3 [file 13012_2023_1319_MOESM3_ESM.docx]
